# Comparative Efficacy and Renal Safety of Entecavir and Tenofovir Disoproxil Fumarate in the Treatment of Chronic Hepatitis B: A Retrospective Cohort Study From Vietnam

**DOI:** 10.1002/prp2.70177

**Published:** 2025-10-02

**Authors:** Thong Duy Vo, Han Ngoc Gia Nguyen, Sang The Phan

**Affiliations:** ^1^ Department of Internal Medicine, School of Medicine University of Medicine and Pharmacy at Ho Chi Minh City Ho Chi Minh City Vietnam; ^2^ Department of Gastroenterology University Medical Center Ho Chi Minh City Ho Chi Minh City Vietnam; ^3^ Department of Clinical Pharmacy, School of Pharmacy University of Medicine and Pharmacy at Ho Chi Minh City Ho Chi Minh City Vietnam

**Keywords:** ALT normalization, chronic hepatitis B, entecavir, fibrosis, renal safety, tenofovir disoproxil fumarate, Vietnam

## Abstract

Tenofovir disoproxil fumarate (TDF) and entecavir (ETV) are first‐line antiviral agents for chronic hepatitis B (CHB), yet comparative real‐world data in Southeast Asian populations remain limited. This retrospective cohort study aimed to compare the efficacy, biochemical response, antifibrotic effect, and renal safety of TDF versus ETV in treatment‐naïve Vietnamese patients with CHB over a 48‐week period. A total of 348 patients (TDF: 181; ETV: 167) were included and evaluated at baseline, Weeks 12, 24, and 48. Both groups demonstrated comparable virologic suppression at Week 48 (TDF: 58.0%, ETV: 52.1%, *p* > 0.05). TDF achieved significantly higher ALT normalization at Week 24 (72.9% vs. 59.9%, *p* = 0.01) and Week 48 (84.0% vs. 69.5%, *p* = 0.001). In contrast, ETV led to faster AST normalization and greater early reductions in APRI and FIB‐4 at Week 12. Renal function mildly declined in the TDF group (mean eGFR change: −3.84 ± 11.98, *p* < 0.001) but improved in the ETV group (+3.02 ± 12.34, *p* = 0.002). Both treatments were well tolerated with no virologic breakthrough or serious adverse events. In conclusion, both TDF and ETV offer effective antiviral therapy for Vietnamese CHB patients. TDF may be preferable in patients with active hepatic inflammation, whereas ETV may benefit those with baseline renal concerns or early fibrotic progression. These findings support a tailored approach to HBV management based on individual patient profiles.

## 1 Introduction

Chronic hepatitis B virus (HBV) infection remains a significant global health concern, with an estimated 296 million people chronically infected and 820,000 deaths annually from HBV‐related complications such as cirrhosis and hepatocellular carcinoma (HCC) [1]. The World Health Organization (WHO) and international liver societies recommend long‐term antiviral therapy to suppress viral replication and prevent disease progression [2, 3]. Among the first‐line agents, tenofovir disoproxil fumarate (TDF) and entecavir (ETV) are the most widely used nucleos(t)ide analogues, demonstrating potent antiviral activity and high genetic barriers to resistance [4, 5].

Several studies have reported comparable efficacy between TDF and ETV in terms of HBV DNA suppression, biochemical response, and HBeAg seroconversion [6, 7]. However, real‐world data suggest differences in biochemical normalization rates, renal safety profiles, and potential long‐term outcomes including fibrosis regression and HCC prevention [8, 9]. In Vietnam, where the burden of chronic HBV infection remains high, especially among adults born before the introduction of the HBV vaccine, understanding local treatment outcomes is critical to optimizing therapeutic strategies [10]. Recent studies from Vietnam, including those by Vo and colleagues, emphasize the need for population‐specific data to support clinical decision‐making in HBV management [11, 12].

This study aims to compare the efficacy and safety of TDF versus ETV over 48 weeks in treatment‐naïve Vietnamese patients with chronic hepatitis B, focusing on viral, biochemical, fibrotic, and renal outcomes.

## 2 Materials and Methods

### 2.1 Study Design and Setting

This was a single‐center, retrospective cohort study conducted at the University Medical Center, University of Medicine and Pharmacy at Ho Chi Minh City, Vietnam. The hospital is a major tertiary referral center serving a diverse population in southern Vietnam. The study aimed to evaluate and compare the efficacy and renal safety of entecavir (ETV) and tenofovir disoproxil fumarate (TDF) in treatment‐naïve patients with chronic hepatitis B (CHB).

### 2.2 Study Population

Eligible patients were adults (≥ 18 years) with CHB, defined as hepatitis B surface antigen (HBsAg) positivity for more than 6 months, who were initiated on monotherapy with either ETV (0.5 mg daily) or TDF (300 mg daily) between January 1, 2018, and January 1, 2023. Patients were included if they had complete clinical and laboratory data at baseline and at 12, 24, and 48 weeks of follow‐up. Exclusion criteria included: co‐infection with hepatitis C virus (HCV), hepatitis D virus (HDV), or human immunodeficiency virus (HIV); evidence of cirrhosis or hepatocellular carcinoma (HCC) at baseline; alcohol‐related liver disease; drug‐induced liver injury; and metabolic‐associated fatty liver disease (MAFLD). Patients with missing key laboratory data or those lost to follow‐up before Week 48 were also excluded.

### 2.3 Data Collection and Outcomes

Data were extracted from electronic medical records, including demographics, comorbidities, HBeAg status, HBV DNA levels, alanine aminotransferase (ALT), aspartate aminotransferase (AST), serum creatinine, and platelet count. The AST‐to‐Platelet Ratio Index (APRI) and Fibrosis‐4 (FIB‐4) scores were calculated to estimate liver fibrosis. Estimated glomerular filtration rate (eGFR) was calculated using the CKD‐EPI formula.

Primary efficacy endpoints included virologic response (undetectable HBV DNA), HBeAg seroconversion, and normalization of ALT and AST. Secondary endpoints included changes in APRI and FIB‐4 scores and renal safety outcomes (change in serum creatinine and eGFR).

### 2.4 Statistical Analysis

Descriptive statistics were used to summarize patient characteristics and treatment outcomes. Continuous variables were presented as means ± standard deviation (SD) or medians with interquartile range (IQR), and compared using the Student's *t*‐test or Mann–Whitney *U* test, as appropriate. Categorical variables were expressed as frequencies and percentages, and compared using the chi‐square test or Fisher's exact test. Repeated‐measures analysis and paired comparisons were performed for intra‐group changes over time. Kaplan–Meier survival curves and log‐rank tests were used to compare the time to ALT normalization between groups. All statistical analyses were conducted using SPSS version 26.0 (IBM Corp., Armonk, NY, USA). A two‐sided *p*‐value < 0.05 was considered statistically significant.

## 3 Results

### 3.1 Baseline Characteristics

A total of 348 treatment‐naïve patients with chronic hepatitis B were included in this study. Of these, 181 patients received tenofovir disoproxil fumarate (TDF) and 167 patients received entecavir (ETV). The median age was 45.5 years (IQR: 36–54), and 56.1% of the study population was male. Baseline demographic and clinical characteristics were comparable between the two treatment groups, with no statistically significant differences in age, sex distribution, eGFR, serum creatinine, HBeAg status, baseline HBV DNA levels, ALT, AST, or liver fibrosis indices (APRI and FIB‐4) (all *p* > 0.05) (Table 1).

**TABLE 1:** Baseline characteristics of the study population.

Variable	Total (*n* = 348)	TDF group (*n* = 181)	ETV group (*n* = 167)	*p*
Age, median (IQR)	45.5 (36–54)	46 (36–53)	45 (35–58)	0.649
Male, *n* (%)	200 (56.1)	106 (58.6)	94 (56.3)	0.668
eGFR (mL/min/1.73 m^2^), mean ± SD	82.4 ± 14.2	83.8 ± 11.4	81.0 ± 16.6	0.063
Serum creatinine (mg/dL), mean ± SD	0.91 ± 0.16	0.89 ± 0.01	0.93 ± 0.01	0.069
APRI score, mean ± SD	0.63 ± 0.02	0.62 ± 0.03	0.64 ± 0.04	0.540
FIB‐4 index, mean ± SD	1.44 ± 0.05	1.36 ± 0.06	1.52 ± 0.09	0.986
HBeAg positive, *n* (%)	193 (55.5)	116 (64.1)	77 (46.1)	0.295
HBV DNA (log10 IU/mL), median (IQR)	6.24 (3.85–7.98)	6.43 (4.17–7.98)	5.90 (3.50–7.98)	0.188

### 3.2 Virologic and Serologic Response

Virologic suppression and HBeAg seroconversion were assessed at 12, 24, and 48 weeks. At week 12, 18 patients (9.9%) in the TDF group and 23 patients (13.8%) in the ETV group achieved HBV DNA levels below the limit of detection. By Week 24, the rates of undetectable HBV DNA were comparable between the two groups: 27.6% in TDF vs. 27.5% in ETV. At Week 48, virologic response was observed in 58.0% of TDF‐treated patients and 52.1% of ETV‐treated patients. No statistically significant difference was observed in virologic response between the two groups at any time point (*p* > 0.05) (Table 2).

**TABLE 2:** Proportion of patients achieving undetectable HBV DNA.

Timepoint	TDF group (*n* = 181)	ETV group (*n* = 167)	*p*
Week 12	18 (9.9%)	23 (13.8%)	> 0.05
Week 24	50 (27.6%)	46 (27.5%)	> 0.05
Week 48	105 (58.0%)	87 (52.1%)	> 0.05

Among HBeAg‐positive patients at baseline (*n* = 193), seroconversion was observed in 2.6% of patients in both groups at Week 12. At Week 24, seroconversion rates were 5.2% in the TDF group and 6.5% in the ETV group. By Week 48, the TDF group showed a higher seroconversion rate (18.1%) compared to the ETV group (9.1%). However, the differences at all time points were not statistically significant (*p* > 0.05) (Table 3).

**TABLE 3:** HBeAg seroconversion in HBeAg‐positive patients.

Timepoint	TDF group (*n* = 116)	ETV group (*n* = 77)	*p*
Week 12	3 (2.6%)	2 (2.6%)	> 0.05
Week 24	6 (5.2%)	5 (6.5%)	> 0.05
Week 48	21 (18.1%)	7 (9.1%)	> 0.05

### 3.3 Biochemical Response: ALT and AST Normalization

**FIGURE 1:**
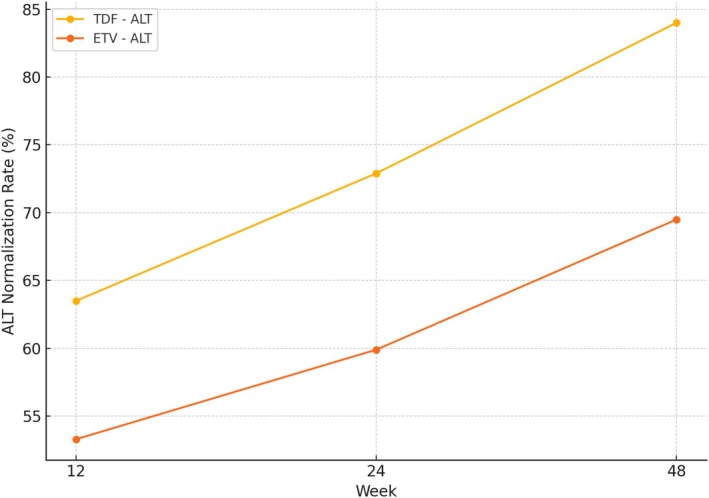
ALT normalization rates at Weeks 12, 24, and 48.

Normalization of alanine aminotransferase (ALT) and aspartate aminotransferase (AST) levels was evaluated at Weeks 12, 24, and 48. At Week 12, ALT normalization occurred in 63.5% of patients in the TDF group compared to 53.3% in the ETV group (*p* > 0.05). By Week 24, ALT normalization was significantly more frequent in the TDF group (72.9%) than in the ETV group (59.9%, *p* = 0.01). This trend remained at Week 48, where 84.0% of patients receiving TDF achieved ALT normalization versus 69.5% in the ETV group (*p* = 0.001) (Figure 1).

**FIGURE 2:**
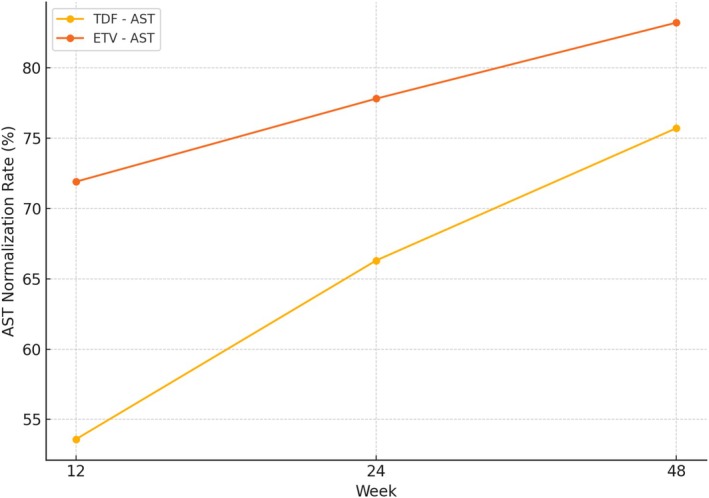
AST normalization rates at Weeks 12, 24, and 48.

In contrast, AST normalization was consistently more frequent in the ETV group across all timepoints. At Week 12, 71.9% of ETV‐treated patients had normalized AST levels versus 53.6% in the TDF group (*p* < 0.05). At Week 24, AST normalization remained higher in the ETV group (77.8%) compared to the TDF group (66.3%, *p* < 0.05). By Week 48, normalization of AST was observed in 83.2% of patients in the ETV group and 75.7% in the TDF group (*p* < 0.05) (Figure 2).

### 3.4 Liver Fibrosis Markers: APRI and FIB‐4

The APRI and FIB‐4 indices were used as non‐invasive surrogate markers to evaluate changes in liver fibrosis over time.

At Week 12, the mean APRI score decreased from 0.62 ± 0.03 to 0.48 ± 0.02 in the TDF group and from 0.64 ± 0.04 to 0.44 ± 0.02 in the ETV group. The absolute reduction in APRI was significantly greater in the ETV group (−0.20 ± 0.31) compared to the TDF group (−0.14 ± 0.29, *p* = 0.008). This trend continued at Week 24, although the between‐group difference was no longer statistically significant. By Week 48, APRI scores were similarly reduced in both groups (0.39 ± 0.01 vs. 0.41 ± 0.02, *p* = 0.457) (Table 4).

**TABLE 4:** APRI score and reduction from baseline.

Timepoint	Group	APRI (Mean ± SD)	Δ from baseline	Intra‐group *p*	Inter‐group *p*
Baseline	TDF	0.62 ± 0.03	—	—	0.540
	ETV	0.64 ± 0.04	—	—	
Week 12	TDF	0.48 ± 0.02	−0.14 ± 0.29	< 0.001	*0.008*
	ETV	0.44 ± 0.02	−0.20 ± 0.31	< 0.001	
Week 24	TDF	0.43 ± 0.02	−0.19 ± 0.31	< 0.001	0.167
	ETV	0.42 ± 0.02	−0.22 ± 0.31	< 0.001	
Week 48	TDF	0.39 ± 0.01	−0.23 ± 0.32	< 0.001	0.457
	ETV	0.41 ± 0.02	−0.23 ± 0.31	< 0.001	

Regarding FIB‐4, patients in the TDF group demonstrated a gradual reduction from 1.36 ± 0.06 at baseline to 1.22 ± 0.06 at Week 48 (Δ = −0.14 ± 0.67, *p* = 0.002). In contrast, the ETV group showed a steeper decline at Week 12 (Δ = −0.23 ± 0.58), reaching 1.29 ± 0.08, followed by stabilization at Weeks 24 and 48. No statistically significant difference in FIB‐4 values was observed between the two groups at Week 48 (*p* = 0.480) (Table 5).

**TABLE 5:** FIB‐4 Score and Reduction from Baseline.

Timepoint	Group	FIB‐4 (mean ± SD)	Δ from baseline	Intra‐group *p*	Inter‐group *p*
Baseline	TDF	1.36 ± 0.06	—	—	0.986
	ETV	1.52 ± 0.09	—	—	
Week 12	TDF	1.33 ± 0.06	−0.03 ± 0.50	0.267	0.147
	ETV	1.29 ± 0.08	−0.23 ± 0.58	< 0.001	
Week 24	TDF	1.29 ± 0.06	−0.07 ± 0.56	0.042	0.403
	ETV	1.30 ± 0.08	−0.22 ± 0.66	< 0.001	
Week 48	TDF	1.22 ± 0.06	−0.14 ± 0.67	0.002	0.480
	ETV	1.35 ± 0.09	−0.17 ± 0.92	0.020	

Table 5 presents the FIB‐4 index progression over 48 weeks. At baseline, the mean FIB‐4 score was 1.36 ± 0.06 in the TDF group and 1.52 ± 0.09 in the ETV group (*p* = 0.986). At Week 12, the ETV group showed a steeper decline in FIB‐4 score (−0.23 ± 0.58) compared to the TDF group (−0.03 ± 0.50), with a significant within‐group difference in the ETV arm (*p* < 0.001). The difference between groups, however, was not statistically significant (*p* = 0.147). At Week 24, both groups showed further improvement with no significant inter‐group difference (*p* = 0.403). By Week 48, the TDF group had reduced their FIB‐4 to 1.22 ± 0.06, while the ETV group returned to 1.35 ± 0.09. Although intra‐group changes remained statistically significant, the between‐group difference was not (*p* = 0.480).

### 3.5 Renal Safety: Serum Creatinine and eGFR

Renal function was evaluated using serum creatinine and estimated glomerular filtration rate (eGFR) at baseline and at Weeks 12, 24, and 48. In the TDF group, mean serum creatinine increased from 0.89 ± 0.01 mg/dL at baseline to 0.93 ± 0.01 mg/dL at Week 48 (Δ = +0.03 ± 0.11, *p* < 0.001). In contrast, the ETV group experienced a slight decrease from 0.93 ± 0.01 mg/dL to 0.90 ± 0.01 mg/dL (Δ = −0.02 ± 0.11, *p* = 0.004). The between‐group difference in creatinine levels was not statistically significant at Week 48 (*p* = 0.178) (Table 6). Similarly, the eGFR declined steadily in the TDF group from 83.8 ± 0.85 to 80.0 ± 0.97 mL/min/1.73 m^2^ (Δ = −3.84 ± 11.98, *p* < 0.001), while it improved in the ETV group from 80.0 ± 1.28 to 84.0 ± 1.24 mL/min/1.73 m^2^ (Δ = +3.02 ± 12.34, *p* = 0.002). The inter‐group differences in eGFR change were statistically significant at both Week 24 (*p* = 0.007) and Week 48 (*p* = 0.010) (Table 7).

**TABLE 6:** Changes in serum creatinine over 48 weeks.

Timepoint	Group	Creatinine (mg/dL)	Δ from baseline	Intra‐group *p*	Inter‐group *p*
Baseline	TDF	0.89 ± 0.01	—	—	0.069
	ETV	0.93 ± 0.01	—	—	
Week 12	TDF	0.92 ± 0.01	+0.02 ± 0.10	0.002	0.625
	ETV	0.92 ± 0.02	−0.01 ± 0.10	0.280	
Week 24	TDF	0.92 ± 0.01	+0.03 ± 0.11	< 0.001	0.403
	ETV	0.90 ± 0.01	−0.03 ± 0.10	< 0.001	
Week 48	TDF	0.93 ± 0.01	+0.03 ± 0.11	< 0.001	0.178
	ETV	0.90 ± 0.01	−0.02 ± 0.11	0.004	

**TABLE 7:** Changes in estimated glomerular filtration rate (eGFR).

Timepoint	Group	eGFR (mL/min/1.73 m^2^)	Δ from baseline	Intra‐group *p*	Inter‐group *p*
Baseline	TDF	83.8 ± 0.85	—	—	0.063
	ETV	80.0 ± 1.28	—	—	
Week 12	TDF	80.3 ± 0.90	−3.46 ± 7.71	< 0.001	0.195
	ETV	82.4 ± 1.25	+1.47 ± 8.33	0.024	
Week 24	TDF	80.3 ± 1.00	−3.50 ± 11.61	< 0.001	0.007
	ETV	83.7 ± 1.20	+2.76 ± 11.20	0.002	
Week 48	TDF	80.0 ± 0.97	−3.84 ± 11.98	< 0.001	0.010
	ETV	84.0 ± 1.24	+3.02 ± 12.34	0.002	

### 3.6 Adverse Events and Drug Discontinuation

Adverse events were generally mild and manageable in both treatment groups. No patient experienced a severe or life‐threatening complication during the study period. The most common adverse event reported was fatigue, occurring in 10.5% of patients in the TDF group and 9.0% in the ETV group. Gastrointestinal discomfort (e.g., nausea or abdominal bloating) was noted in 8.3% of TDF‐treated patients and 6.0% of ETV‐treated patients. Mild headaches were documented in 5.5% and 4.8% of patients in the TDF and ETV groups, respectively. Skin rashes and transient elevations in transaminases were uncommon and not significantly different between groups (Table 8).

**TABLE 8:** Summary of adverse events.

Adverse event	TDF group (*n* = 181)	ETV group (*n* = 167)	*p*
Fatigue	19 (10.5%)	15 (9.0%)	> 0.05
GI discomfort	15 (8.3%)	10 (6.0%)	> 0.05
Headache	10 (5.5%)	8 (4.8%)	> 0.05
Skin rash	3 (1.7%)	2 (1.2%)	> 0.05
Elevated transaminases	2 (1.1%)	1 (0.6%)	> 0.05
Drug discontinuation	2 (1.1%)	0 (0.0%)	> 0.05

Regarding drug discontinuation, only two patients in the TDF group (1.1%) discontinued treatment due to increasing creatinine levels. No patients in the ETV group discontinued therapy due to adverse events. No virological breakthroughs or drug resistance mutations were detected during the study.

## 4 Discussion

This real‐world study presents a head‐to‐head comparison of tenofovir disoproxil fumarate (TDF) and entecavir (ETV) over 48 weeks in treatment‐naïve Vietnamese patients with chronic hepatitis B (CHB). Our findings demonstrate that both antivirals are effective in achieving virologic suppression and HBeAg seroconversion, consistent with international trials and cohort studies [4, 6, 7]. Importantly, our study provides updated local evidence for clinical decision‐making in Southeast Asia, where HBV remains endemic and treatment data are scarce [11, 12]. A key observation was the divergence in biochemical responses. TDF achieved significantly higher ALT normalization at Weeks 24 and 48, supporting its role in reducing hepatic inflammation [8]. Possible mechanistic explanations may include more rapid intracellular suppression of viral replication or pharmacodynamic differences between TDF and ETV. However, these mechanisms remain speculative and warrant further mechanistic and translational studies. Conversely, ETV resulted in earlier AST normalization, suggesting differential effects on hepatocellular injury pathways. These findings mirror patterns seen in previous Asian cohorts [7, 9]. To further interpret these findings, earlier AST normalization with ETV may reflect a faster resolution of hepatocellular injury. Clinically, this could be relevant in patients where early biochemical recovery is critical, such as those with comorbidities or at risk of hepatic decompensation.

Fibrosis marker trends were also notable. ETV led to faster early reductions in APRI and FIB‐4 at Week 12, while long‐term fibrosis improvement at Week 48 was similar between both groups. These results suggest that although short‐term antifibrotic responses may differ, sustained therapy with either agent yields comparable structural liver benefit [6, 13]. Renal safety remains a differentiating factor. As reported in other global and Asian studies [8, 14], TDF was associated with mild declines in eGFR, whereas ETV showed stable or improved renal function. Our findings are consistent with complementary real‐world data reported by Akdemir Kalkan et al. [15], which demonstrated improved renal safety outcomes when switching from TDF to TAF or ETV. This distinction may inform individualized therapy, especially for patients with borderline renal reserve or older age groups [12]. To further highlight the clinical implications, we emphasize that TDF may be prioritized in patients with marked hepatic inflammation, while ETV should be considered in those with impaired renal reserve or comorbid conditions. These distinctions underscore the importance of individualized antiviral selection in real‐world practice. Both regimens were well tolerated, with low rates of adverse events and discontinuation. No virologic breakthroughs or resistance were observed over 48 weeks, reinforcing the durability of these first‐line agents in the Vietnamese population.

The strengths of our study include its real‐world setting, balanced cohort size, uniform monitoring intervals, and multi‐dimensional assessment (virologic, biochemical, fibrotic, renal). Limitations include its retrospective design, single‐center nature, and 48‐week duration. Nonetheless, it addresses a key gap in regional HBV management and contributes valuable comparative insights for frontline therapy.

## 5 Conclusion

Both tenofovir disoproxil fumarate (TDF) and entecavir (ETV) demonstrated excellent efficacy and tolerability over 48 weeks in treatment‐naïve Vietnamese patients with chronic hepatitis B. However, the follow‐up in our study was limited to 48 weeks, which may not fully capture longer‐term antiviral efficacy and safety outcomes. TDF was superior in ALT normalization, while ETV offered earlier antifibrotic effects and a more favorable renal safety profile. These distinctions support the need for personalized antiviral selection based on patient comorbidities and treatment goals. Our findings affirm current international guidelines while highlighting region‐specific insights. Further prospective and long‐term multicenter studies are warranted to validate these findings, particularly regarding outcomes such as cirrhosis regression and hepatocellular carcinoma prevention. Including data from underrepresented populations such as Vietnam is essential to advancing equitable, evidence‐based HBV care globally.

## Author Contributions

T.D.V. and H.N.G.N. contributed to the study conception, design, data collection, analysis, and manuscript writing. T.D.V., H.N.G.N., and S.T.P. revised the manuscript. T.D.V. supervised the study and critically revised the manuscript. All authors reviewed and approved the final version of the manuscript.

## Ethics Statement

This study was approved by the Institutional Review Board of the University Medical Center, Ho Chi Minh City (Approval No. 862/HĐĐĐ‐ĐHYD, dated September 28, 2023). As the study was retrospective and used anonymized patient data, the requirement for informed consent was waived.

## Conflicts of Interest

The authors declare no conflicts of interest.

## Data Availability

The data that support the findings of this study are available from the corresponding author upon reasonable request.
